# Baseball Related Injuries: A Case Report on Acute Compartment Syndrome of the Forearm

**DOI:** 10.1155/2022/5449913

**Published:** 2022-03-06

**Authors:** Charles B. Pasque, Christopher Hendrix

**Affiliations:** Department of Orthopaedic Surgery and Rehabilitation, University of Oklahoma College of Medicine, Oklahoma City, Oklahoma, USA

## Abstract

Acute compartment syndrome is a difficult diagnosis to make due to its wide range of clinical presentations. Delay or misdiagnosis can cause devastating consequences such as Volkmann's ischemic contracture, permanent nerve damage, amputation, and death. Lower extremity compartment syndrome is more common than upper extremity compartment syndrome, with the forearm being the most common location for upper extremity compartment syndrome. Acute compartment syndrome is most caused by acute fracture trauma but can also be due to soft tissue crush injuries or vascular problems. We report a unique case of a male umpire being struck on the forearm by a baseball with subsequent progression to an acute compartment syndrome that required emergent fasciotomies. The patient made a full recovery with no known long-term sequelae.

## 1. Introduction

Acute compartment syndrome of the extremity occurs when the interstitial pressure of the tissues exceeds the capillary perfusion pressure either from bleeding or edema in the compartment [1]. This can result from external pressure on the compartment or decreased arterial inflow [2]. The majority of compartment syndromes occur as a result of high-energy trauma with an accompanying fracture. Around one-fourth of forearm compartment syndromes occur because of soft tissue injury alone, and in 10 percent of these cases, the patient has an underlying coagulopathy or known bleeding disorder [3]. Acute compartment syndrome is largely a clinical diagnosis, and it is vitally important that the health-care provider recognizes it within the appropriate period. Acute compartment syndrome is typically diagnosed by using somof the five “P's,” which includes pain, paresthesia, paralysis, pallor, and pulselessness. The outcome of any acute compartment syndrome significantly hinges on the time elapsed from the onset of pathology to the diagnosis and subsequent compartmental release. In general, irreversible damage occurs after eight hours has elapsed from the onset of ischemia [4–7]. We present a case of acute volar and dorsal compartment syndrome of the forearm after an impact injury during a baseball game. To our knowledge, this is the first case report in the literature of a compartment syndrome after being struck by a baseball.

## 2. Narrative

A 51-year-old right-hand dominant male and home plate baseball umpire was struck on the right proximal dorsal forearm approximately three hours prior to his presentation to the Emergency Department. The patient stated that he was struck during the first game of a double-header and continued to umpire while leaving his right arm in a dependent position. He subsequently developed progressive worsening pain and paresthesia in his right forearm and hand. The patient had no other medical problems and was taking no medications.

On the initial physical exam, the patient's right upper extremity had a swollen dorsal forearm over the mobile extensor muscle wad. The patient was able to flex and extend his fingers with a painless passive arc of motion. His sensation was slightly decreased in the sensory radial nerve distribution but fully intact in the median and ulnar nerve distribution. The patient was sent for X-ray, which was negative for fracture. On return from X-ray, his repeat exam revealed tense compartments, pain with passive stretch causing nausea, absent sensation in the radial nerve distribution, and decreased sensation in the median nerve distribution. The dorsal forearm compartment pressures were measured in the emergency department using the Quick Pressure Monitor System (Stryker Instruments, Kalamazoo, MI, U.S.A.). The dorsal compartment measured 45 mmHg and the volar compartment measured 30 mmHg. The patient's coagulation studies were normal.

The patient was taken to the operating room for immediate forearm fasciotomies. Under general anesthesia, a dorsal compartment release was performed through a midline dorsal incision. The dorsal mobile extensor muscle wad was released. The muscles appeared very swollen without necrosis evident. The volar fasciotomy was performed with decompression of the superficial and deep volar compartments. The muscles were minimally swollen and there was a small hematoma within the deep volar compartment. The fasciotomy incisions were left open after the initial surgery. The patient required two more trips to the operating room over the following seven days for repeat irrigation and debridement to allow the swelling to improve for delayed primary closure of his incisions. The median nerve sensation returned to normal after the initial fasciotomy surgery.

On discharge, the patient was placed in a soft dressing and started on range of motion exercises. At four weeks postoperatively, the patient had no pain and the paresthesia in the sensory radial nerve had resolved. At three months, the patient had regained full range of motion and strength in his forearm without any pain.

## 3. Discussion

Compartment syndrome is one of the true orthopedic emergencies and occurs due to bleeding or edema within a closed osteofascial compartment. This leads to compression of the veins and a decreased arteriovenous pressure gradient, ultimately resulting in ischemia [1, 2, 4, 6]. Tissue fascia is avascular and nonelastic, making it resistant to acute stretching and resulting in fixed spaces [8]. If the period from the onset of ischemia to compartment release is within four hours, complete resolution is most often seen. Damage is variable between six to eight hours and irreversible damage can occur after eight hours [4]. Compartment syndrome remains largely a clinical diagnosis and is often difficult to recognize due to the variety of etiologies and injury patterns. Swollen and tense compartments are sometimes an obvious and important clinical finding. In addition, the five “P's” are historically used when making the diagnosis and include pain (out of proportion), paresthesia, paralysis, pallor, and pulselessness. Pain with passive stretching is often used as the sixth “P,” and along with pain out of proportion, they are the first and most sensitive signs of acute compartment syndrome [1–4, 6, 8]. Pallor and pulselessness are not normally present on initial presentation and typically are a sign of late-stage compartment syndrome and probable irreversible damage [4, 9]. A critical analysis review found that risk factors for a delay in diagnosis included relying only on clinical signs, having an associated neurological injury, acute compartment syndrome in children or a patient with reduced or altered consciousness, regional or patient-controlled analgesia, and lack of experience of evaluating medical personnel [10].

As soon as the diagnosis of acute compartment syndrome is made, a fasciotomy should be performed to prevent the devastating complications of Volkmann's ischemic contracture, neurologic deficits, infection, amputation, or rarely death [6]. Volkmann's ischemic contracture is usually the chronic end stage and is the result of replacement of the compartment's muscle tissue with fibrotic tissue, causing contracture and nerve dysfunction [5]. The deep flexor muscles are more susceptible to compression and ischemic injury due to their fascial boundaries preventing expansion [4, 8]. The median nerve is the nerve most often injured in forearm compartment syndrome, lying between the flexor digitorum superficialis and the flexor digitorum profundus [8].

There have been many different recommendations over time regarding thresholds of tissue pressure indicating a need for fasciotomy. Normal resting tissue pressure is within 0-10 mmHg [6, 8]. The most widely accepted indication is a tissue pressure that is within 10-30 mmHg of the diastolic blood pressure, known as the delta pressure [2, 3, 5–11]. An absolute pressure greater than 30 mmHg is also an indication for fasciotomy, especially when other clinical signs are present [3, 5–8]. A concerning physical exam and an absolute pressure of greater than 20 mmHg are accepted as an indication to proceed to compartment release [3]. Diastolic pressure is often used as a guide due to hypotensive patients being more prone and hypertensive patients being less prone to compartment syndrome [4, 9]; thus, hypertension can be a protective factor and may be another reason older patients are less commonly affected [12].

A study published in 2019 found that the relative risk of morbidity and mortality is higher in those treated with fasciotomy than with a nonsurgical approach [13]. However, another recent study stated that a nonsurgical approach is not recommended, and patients successfully treated nonsurgically likely did not have true compartment syndrome after all [8]. Above all, the health-care provider must remember that these are only clinical guidelines and that the ultimate decision-making is up to the knowledge and experience of the health-care provider.

There are three different forms of compartment syndrome which are acute, chronic, and neonatal. Chronic is otherwise known as chronic exertional compartment syndrome and is characterized by recurring, transient increases in tissue pressure, usually during exercise, causing pain and neurologic symptoms that resolve with rest [5]. Neonatal is extremely rare and occurs within the first 24 hours of life. It is usually identified by swelling and discoloration of the limb and is caused by an unknown pathophysiology [14]. Acute compartment syndrome in adults most commonly affects males, with an incidence ten times greater when compared to females [15]. The higher incidence in males is thought to be due to their larger muscle mass within a fixed compartment, and that males are more likely involved in high-energy trauma [6, 12]. In adults and children, the most common location of acute compartment syndrome is the lower extremity and is usually due to a fracture. The most common location of fracture to cause acute compartment syndrome occurs in the tibia and distal radius [7]. In the upper extremity, it is estimated that 1.22 percent of all forearm fractures require fasciotomy [16]. Distal radius fractures are the most common cause of acute compartment syndrome of the forearm in adults, while supracondylar fractures are the most common cause in children. The second most common overall cause of acute compartment syndrome is soft tissue related, making up an estimated 23 percent of all cases [3, 8, 10]. Soft tissue injuries can be subdivided into crush injuries, burns, edema, intramuscular hematoma, snake bites, drug, or alcohol overuse induced stupor, with these causes being more common in patients with an underlying coagulopathy or known bleeding disorder [7, 15]. A relatively rare cause includes high-pressure injection injury to the upper extremity, such as by gunshot and pressurized gas and liquids, with the overwhelming majority occurring via occupational injury [17]. Other factors associated with forearm compartment syndrome secondary to soft tissue injury include increased age, medical comorbidities, and anticoagulation [10]. While 10 percent of soft tissue injuries resulting in compartment syndrome have a coagulopathy or known bleeding disorder [3], our patient in this case report's coagulation studies was normal.

Due to baseball's widespread popularity, it has one of the highest numbers of Emergency Department visits. The number of visits is likely underestimated because it does not include physician visits outside of the Emergency Department [18]. Baseball-related injuries have significantly declined over the last twenty years due to increased safety measures and precautions. The upper extremity is the second most common body part injured trailing only the face, with most being a soft tissue injury after being hit by a baseball [19]. This is one of two reports in the literature of an acute forearm compartment syndrome after being struck by a ball without a fracture. The other such report was published in 2006 of a man suffering from three separate acute compartment syndromes from minor trauma and without a fracture over a seven-year span [20]. The patient's coagulation studies were normal, except for one abnormal bleeding time after his first acute compartment syndrome case. The authors hypothesized that he may have had an underlying variant of Von Willebrand's disease.

The patient in our case report presented with elevated compartment pressures of 45 mmHg in the dorsal compartment and 30 mmHg in the volar compartment. The patient had volar and dorsal compartments that were tense, pain with passive motion of the fingers, paresthesia, and pain out of proportion to his injury. All these findings were consistent with an acute forearm compartment syndrome. The patient's volar, dorsal, and mobile wad compartments were all released, and the patient's symptoms improved gradually over the next four weeks.

## 4. Conclusion

This case report highlights that acute upper extremity compartment syndrome can develop after a major blow to the forearm soft tissues with high velocity objects, such as a baseball that may not result in bone fractures or obvious soft tissue damage on initial impact. This emphasizes the importance of complete and thorough serial physical examination, even after an initially benign-appearing injury. All health-care providers at athletic events and Emergency Departments must be aware of the potential for compartment syndrome after blows from an object such as a baseball. In addition, maintaining a high level of clinical suspicion and diligence is important to make a timely diagnosis and treatment that will provide the patient with the best possible outcome. To our knowledge, this is the first case report of an acute forearm compartment syndrome during a sporting event due to a blow by a baseball.

## Data Availability

No data are associated with this article.
